# Trauma Triage and Trauma System Performance

**DOI:** 10.5811/westjem.2016.2.29900

**Published:** 2016-04-26

**Authors:** Gary Johnson

**Affiliations:** University Hospital, SUNY Upstate Medical University, Department of Emergency Medicine, Syracuse, New York

Trauma systems seek to provide complex medical care at the correct time and the correct place. During the past four decades numerous articles have been published that validate trauma systems from many points of view. Achievements of trauma systems include improvement in mortality and morbidity, efficiencies of care, and economic outcomes. Prehospital policy execution is intrinsic to trauma system performance. Trauma system criteria are relatively standardized. However, flexibility in emergency medical service (EMS) decision-making is commonly allowed. These decisions have major impacts on resource allocation, trauma center utilization, and patient outcome.

In this edition, Holst, et al^1^ reviewed adult emergency department (ED) trauma deaths as reported in the 2010 National Emergency Department Sample. They recorded the association of these deaths to trauma or non-trauma center designation, as well as geographic and patient demographics including rural vs urban site, gender, and patient income data. They found that one half of all trauma ED deaths nationally and one third of ED urban trauma patients died in non-trauma centers. Both elderly trauma deaths and deaths due to falls more frequently occurred in non-trauma centers. Like most studies describing trauma system performance, this is a retrospective review taken from a large database. Therefore, causation of outcome cannot be directly attributed to undertriage. However, the magnitude of the non-trauma center death rate merits further investigation.

Trauma system literature often describes the undertriage of trauma patients with regard to trauma center designation. The American College of Surgeons has a goal for trauma systems to achieve less than 5% undertriage. However, studies frequently estimate a much higher rate. Like the study published in this journal, undertriage rates are higher with elderly patients.^2^ Centers for Medicare/Medicaid Services claims also identify a high rate of undertriage, and these non-trauma center visits are associated with worse outcomes. Staudenmayer, et al^3^ found that in California undertriage varied substantially by region. Patient factors such as age greater than 55 years, female sex, greater number of co-morbidities and a fall mechanism led to a higher rate of undertriage.

Overtriage to trauma centers commonly occurs as well and contributes to resource mismatch. Tang, et al^4^ found that a significant number of trauma transfers were discharged within 24 hours. Tarima^5^ found that non-trauma centers were transferring 98% of their trauma patients including those with low Injury Severity Scores (ISSs) and high Glasgow Coma Scale (GCS) scores.

Multiple factors contribute to trauma system formation. Population distribution and medical resource allocation strongly affect system performance. Commonly, injuries happen close to the home of the trauma victim. However, motor vehicle accidents have a higher rate of occurring farther from home. Trauma system volume is associated with improved trauma patient mortality and length of stay. Minei, et al^6^ found that increasing trauma center volume was also associated with more ventilator-free days and less severe organ failure outcomes. This triage process predictably leads to a large number of EDs without significant trauma experience, and hence makes them more likely to transfer out the mild to moderately injured patients they do see. As the cycle repeats, the distribution of trauma care skews even more toward trauma centers. Mohan, et al^7^ confirmed that emergency physicians working in non-trauma centers rarely encounter trauma patients with moderate to severe injuries. Trauma system improvement processes may initially realize gains that are hard to sustain. Winchell, et al^8^ found that trauma system evaluation planning committee consultations commonly improved outcomes. However, the gains were not self-sustaining after consultation.

Trauma system literature is rich with retrospective analyses of trauma system performance. Holst, et al^1^ published in this edition contributes to this literature in a meaningful fashion by looking at all ED trauma deaths rather than trauma center mortality/morbidity alone. The study supports the body of literature that shows undertriage has a significant impact on system performance. Particular attention needs to be paid to the consistent theme of undertriage of elderly trauma and fall-related trauma. Moving forward, each system needs to examine its performance to the community it serves beyond the bounds of the trauma centers themselves. Regional trauma systems need to turn toward prospective validation of outcomes so that system structure and protocols can best serve our populations.
